# The New Era of Blood Transfusion—A Triumph of Surgical Skill

**Published:** 1911-10

**Authors:** 


### Abstracts and Selections.

### The New Era of Blood Transfusion—A Triumph of Surgical Skill.

It is seldom that we read newspaper accounts like the following, which appeared early in the year:

"PHILADELPHIA, February 20.

"With a subject selected from the score or more volunteers who had offered their blood in response to a want advertisement that appeared in a newspaper here this morning, a remarkable transfusion operation was performed tonight in the Polyclinic Hospital. The patient was David Poloco, a poor tailor suffering from internal hemorrhages, whose family had already given all the blood their physicians would allow, and had advertised to give their savings for a volunteer in a last effort to save the life of their relative.

"The subject, who hid his identity under the name of 'Smith,' was a powerful looking man about 26 years old who came to the hospital in a taxicab and is said to be a member of a prominent family in this city.

"The transfusion was permitted to continue for thirty minutes, when the white face of the patient had assumed a rosy hue, while the face of the subject, which at the beginning of the operation was ruddy, had become almost deadly white. An examination of the blood of the two men at the close of the operation showed that the coloring matter in Poloco's blood had risen from 18 to 47 per cent, while that of the subject had decreased from 85 to 69 per cent."

Dr. Crile, of Cleveland, who more than anyone else has *developed* transfusion (the name of experimenters in the past two hundred years is legion), says that he meets with the warmest enthusiasm in asking relatives to make this sacrifice, while Dr. Carrel describes the moral exaltation of a young man who gave his blood to his aged father. It would thus seem that the victory registered over the material is as blood-stirring as the surgical triumph of transfusion itself.

Not that the operation is any longer dangerous. Professional donors of blood have been known to return to hospitals two and three times to repeat the experience; they are often in condition to work the next day. The ordeal is painless. About half an hour beforehand, each patient is given a hypodermic injection of morphine. Local anesthesia is used, a small cut being made over the vessels.

Reducing the surgical history of transfusion into its elements, it began with one Frenchman and was concluded by another; one in the seventeenth, the other in the twentieth century—Jean Denys, who performed the first real operation of the kind; and Alexis Carrel, who found the way to suture the ends of severed blood-vessels.

Denys, through animal experimentation, arrived at conclusions as to the choice of blood-vessels in transfusion which are held valid today. He showed that the artery (as carrier for the quick-flowing red blood) should furnish the supply, and the vein of the sick man should be selected to receive it, as affording the most direct route back to the right heart and the lungs where the blood becomes revitalized. Artery and vein he connected with glass-tube.

He was not sustained by the professional opinion of his time, partly because Harvey's discovery of the circulation, though forty years old, was still ridiculed in Paris, but also in view of the demonstrated negligible quantity of blood capable of being transfused. It had been known for a long time that endothelium, lining arteries and veins (now known as the intima), has the property, practically peculiar to itself, that blood in contact with it does not clot, while, when brought into contact with any other substance, it soon coagulates and ceases to flow.

Denys found that the amount of blood transfused varied according to the size of tube used, and also according to the different size of human and sub-human blood-vessels; though he knew there was no way of satisfactorily gaging the flow save by the pulse of the donor which during the operation must be transmitted from artery to vein. As little as four ounces had brought patients out of syncope, and failure in one case that had promised well. was found on post-mortem to be due to intestinal gangrene.

After initiating the work, he got into trouble. In transfusing blood from the femoral artery of a calf into the veins of an insane man, he was twice successful; but the third trial, made at the insistence of the patient's wife, resulted fatally. Denys was suspicious and wished to open the body. The widow refused, saying he had "experimented" on her husband, and demanding a price for her silence. The doctor's answer was a complaint to the authorities, and in the sequel the French government prohibited the practice.

From this time until early in the nineteenth century this operation was taken up, dropped and taken up at intervals until the method came into vogue of drawing blood from the subject, whipping out the clots (*defibrinating* it) and injecting it into the patient's veins.

Against this method, the great French physiologist, Magendie, gave early warning. His experiments showed that in this way serious symptoms could be made to develop in perfectly healthy animals; and he suspected that the clotting took place now inside the body instead of out, producing paralysis and death. During the Franco-German war, this method was tried on thirty-seven wounded soldiers with only thirteen successes.

A few years later, science began taking those enormous strides which especially in physiology and bio-chemistry have covered more ground than in all preceding history. It was learned that that microscopic body, the white corpuscle, was back of the blood-clot; that when the ends of blood-vessels were cut, it began to manufacture fibrin-ferment. Hence, defibrinating the blood did not solve the problem, as only the solid part of the clot was whipped out and the whole white corpuscle's fibrin-ferment remained in the blood. And it was only when this clotting inside the blood-vessels did not take place in a *vital* organ that Magendie's prognosis was not realized.

Thus the only solution was to join the ends of the blood-vessels together so that, as regarded the circulation, they should become one. But stitches seemed out of the question, since, being foreign bodies, they were as objectionable to the white corpuscles as tubes of glass or paraffine. Yet, if hazardous operations of various kinds ever were to be made safe, to be able to join blood-vessels end-to-end was imperative.

Payr answered this call with a magnesium-tube, but the technique was undeveloped and proved clumsy. Meantime, Carrel was investigating with the blood-vessels of dead human bodies; he applied his technique to living animals, and it worked. He had found the way to do that which everyone had said could not be done, to *sew* the blood-vessels together without clotting or leakage.

The principle of the Carrel method consists in "everting" (turning out) the cut lips of the vessels in such a way that when the openings of the two are brought together, their linings touch. Needle and thread is then drawn through *sterilized vaseline,* passed through the outer and upper edge of the artery in its entire thickness, and brought out through the inner and upper surface of the vein. Two similar stitches are made in the blood-vessels, in all instances leaving about four inches of thread uncut after tying, to serve as a handle for the assistant surgeon to tilt the blood-vessels in order to join them end to end.

Carrel's discovery was hailed with thanksgiving, and he made an early application of it in transfusion by saving the life of a four-day-old baby; this as recently as 1908.

*Dynamics.*—Before Carrel's success, Crile was studying to develop transfusion along mechanical lines. He used Payr's tube, discarded it for Carrel's stitches; and then deciding that the less skillful surgeon needed an easier method, developed the Payr-tube. The instrument he has evolved is a cannula or ring-with-handle, and is so tiny as scarcely to slip over the point of a pencil.

The Crile method is to draw the vein *through* the ring and cuff its edge back *on* the ring; there it is fastened into that one of the two grooves on the ring which happens to be nearest to the handle. The artery is then drawn *over* the veins so its inner surface touches the cuff, and is fastened into the remaining groove. The lining of the two blood-vessels thus meet as in the Carrel suture.

Crile experimented upon hundreds of dogs before operating upon a single human being, and has been roundly abused for his pains by the anti-vivisectionists. There being obvious danger to the giver from anemia, and danger also that the heart of the patient might be filled so full that it could no longer pump, Crile wanted to know just how much blood might be lost and received.

The heart requires a certain volume of fluid (blood) in the vessels in order to have enough to pump on. So, in severe hemorrhage, temporary relief is given by filling up the vessels with salt water (saline-solution, infusion, as it is called). The salt water can not replace the blood, but it gives the heart something with which to pump, and so tides the body over the emergency. After a time, the salt-water leaves the body through the kidneys, and the patient is as badly off as before. This is not the case with transfused blood; that stays in the vessels.

There is another condition, known as "shock," in which, although the blood stays in the vessels, the heart has just as much difficulty to pump as though there had been a bad hemorrhage. The reason is because in shock the injury to the nervous system causes a paralysis of the nerves known as vasomotor. The result of this paralysis is that the blood-pressure disappears just as though a severe hemorrhage had removed the blood, and Crile has shown that, also for this condition, transfusion is the best remedy we have.

A man may be so injured by machinery as to require an immediate operation, but he has lost so much blood that his remaining strength may give way; transfusion rectifies the condition by supplying a sufficient quantity of red blood-cells to combine with the oxygen of the air and give back the oxygen to the tissues. Crile says that recovery from hemorrhage is largely a problem in mechanics, as adrenalin has been added to the circulation even after death, and the heart action substituted by manual pressure—and blood-pressure thus made to rise. We all know when a man is poisoned by gas that the red coloring matter (three hundred times as greedy of carbon-monoxide as of oxygen) has been displaced. Thus the blood is destroyed, and new blood, through transfusion, furnishes a certain corrective, if not delayed too long.

*Research on the Blood.*—Denys in the seventeenth century advocated the use of animal blood in transfusion; maintaining that as the animal lives more closely to a state of nature, his blood must be purer than man's; and answered arguments against it by inquiring why the meat we eat and the milk we drink does not act deleteriously. In view of modern blood-tests, his attitude is instructive as showing what plausible fallacies may be advanced if reasoning, as such, be not corrected by experience.

In 1821, Dumas and Provost gave out the results of their studies on the chemical properties of the blood; showing that when the bloods of different species were mixed, even though the globules were of the same size but of different dimensions, animals rarely lived longer than six days. The blood of an animal with circular corpuscles injected into a bird caused the death of the bird with symptoms of violent poisoning.

In 1875, Landois showed precisely what takes place when the blood is thus mixed. The red corpuscles are dissolved by the poisoning of the protoplasm, and the hemoglobin (coloring matter) exuded from the envelope. Under the microscope, the first stage of this condition (hemolysis) shows the corpuscles beginning to swell. The injection of certain chemicals causes the same phenomenon, and hemolysis occurs on artificial heating or freezing of blood. The consequence is the breaking down of the cell-wall. This objective demonstration, as might have been expected, put a stop to the use of animal blood in transfusion.

Modern transfusion shows that while the blood of one healthy person does not poison the blood of another healthy person, the blood may acquire such power through certain diseases, since the disease may act in such manner as to change it. In this case, too, there is a dissolving of the cells by blood which, though apparently normal, in reality is not. If the sick man's blood has this property of dissolving (hemolyzing) the blood-cells of the giver, the new blood will do him no good, as it will be destroyed as rapidly as it is transfused. If by an oversight the blood of the giver has this property, the transfusion will be even more dangerous, as the new blood will destroy that already present in the patient's vessels. By testing the two bloods against each other this danger may be avoided.

Another peculiar property of the blood is what is called agglutination, known only since 1900. When the bloods of two different persons are mixed, they often have the effect of making the blood-cells clump together in tight little masses. Landsteiner showed that all human beings fall into four sharply defined classes in this regard, and that agglutination may be explained by assuming the existence of two agglutinins, of which the second group possesses one, the third group the other, the first group both, and the fourth neither. When the blood of persons belonging to any one class is mixed with the blood of a person belonging to any of the other three classes, agglutination occurs; but this is avoided if the blood-test shows that the giver and receiver of the blood belong to the same class. This is a hereditary quality, and an examination of seventy-two families shows that the agglutinins follow Mendel's law.

It is evident that if blood were transfused from a person belonging to one of the classes referred to, into the vessels of a person belonging to any one of the other three classes, the clumps likely to form would cause a stopping up of the blood-vessels, and thus serious trouble indeed. Ottenberg is investigating further the phenomenon of agglutination at Columbia College and Mt. Sinai Hospital at the present time. Medical opinion is that performing the agglutination-test for transfusion may, in many cases, make all the difference between a transfusion that is successful and one that means merely a waste of human blood.

Formerly, the patient often became worse after the operation; indiscriminately taking blood from anyone willing to give it was a contributing cause. Now when members of the family are rejected, for the reasons outlined, advertisements are put in the newspapers; fifteen to twenty respondents, at that, often being rejected as unfit to serve, and the one selected whose blood has the least, or no toxic action. The person who gives his blood must be strong enough to lose it, and he must have no blood-disease; by which it would appear that even selling one's blood is not by any means as easy as might be supposed.—Genevieve Grandcourt, in *Scientific American,* August 5, 1911.

